# Post-treatment of secondary wastewater treatment plant effluent using a two-stage fluidized bed bioreactor system

**DOI:** 10.1186/2052-336X-11-10

**Published:** 2013-06-17

**Authors:** Golam Hossein Safari, Kaan Yetilmezsoy, Amir Hossein Mahvi, Mansur Zarrabi

**Affiliations:** 1 Department of Environmental Health Engineering, School of Public Health, Tehran University of Medical Sciences, Tehran, Iran; 2 Department of Environmental Engineering, Faculty of Civil Engineering, Yildiz Technical University, Istanbul, Turkey; 3 Department of Environmental Health Engineering & Center of Water Quality Research, Tehran University of Medical Sciences, Tehran, Iran; 4 Department of Environmental Health Engineering, Faculty of Health, Alborz University of Medical Sciences, Karaj, Iran

**Keywords:** Secondary effluent, COD, Coliform, Fluidized bed reactor

## Abstract

The aim of this study was to investigate the performance of a two-stage fluidized bed reactor (FBR) system for the post-treatment of secondary wastewater treatment plant effluents (Shahrak Gharb, Tehran, Iran). The proposed treatment scheme was evaluated using pilot-scale reactors (106-L of capacity) filled with PVC as the fluidized bed (first stage) and gravel for the filtration purpose (second stage). Aluminum sulfate (30 mg/L) and chlorine (1 mg/L) were used for the coagulation and disinfection of the effluent, respectively. To monitor the performance of the FBR system, variation of several parameters (biochemical oxygen demand (BOD_5_), chemical oxygen demand (COD), turbidity, total phosphorous, total coliform and fecal coliform) were monitored in the effluent wastewater samples. The results showed that the proposed system could effectively reduce BOD_5_ and COD below 1.95 and 4.06 mg/L, respectively. Turbidity of the effluent could be achieved below 0.75 NTU, which was lower than those reported for the disinfection purpose. The total phosphorus was reduced to 0.52 mg/L, which was near the present phosphorous standard for the prevention of eutrophication process. Depending on both microorganism concentration and applied surface loading rates (5–10 m/h), about 35 to 75% and 67 to 97% of coliform were removed without and with the chlorine addition, respectively. Findings of this study clearly confirmed the efficiency of the FBR system for the post-treatment of the secondary wastewater treatment plant effluents without any solid problem during the chlorination.

## Introduction

Application of fluidized bed reactors (FBR) in wastewater treatment has received much attention in the world today due to their high efficiency, and low capital and operating costs. This technology is also gaining popularity as a result of increasingly stringent discharge standards and increased water reclamation demand. In recent years, FBR technology has also been conducted as an effective method to treat various types of high-strength wastewaters such as corn steep liquor [1], distillery effluent [2], synthetic sago wastewater [3], high-sulfate wastewater [4] and so on.

In general, effluents from secondary wastewater treatment plants are further treated in tertiary plants for reuse. In tertiary treatment plants, various methods such as membrane processes, advanced oxidation process, adsorption, filtration and others can be used for purification or post-treatment of wastewater effluents [5,6]. The properties of wastewater effluents can directly influence the filtration rate and selection of the appropriate filter media. In water treatment plants, filter media are often selected as sand matter and fixed in a tank [7]. In filtration of secondary wastewater treatment plant effluents for the future reuse, the filter media sizes are often lighter and greater for prevention from clothing, as well as for providing higher operation time. However, in that case, the filter media may not be able to properly treat the effluents due to greater filter media size [8]. If the filter media is chosen as fine as possible, the clothing and lower operation time will become the main problems. To overcome these problems, a proper alternative can be the treatment of effluents before passing through the filter media [7]. Considering the above-mentioned facts, it is noteworthy that FBR technology can be used as an effective method for the complete biological treatment of the secondary wastewater effluents before the filtration process.

To the best of the authors' knowledge, there are no systematic papers in the literature specifically devoted to a study regarding the application of a two-stage FBR system with a filtration column for treatment of secondary effluents. Therefore, clarification of the place of the present subject in the scheme of secondary wastewater treatment can be considered as a specific field of investigation to compare results with the above-mentioned studies. For that reason, the aim of the present work was to investigate the performance of a two-stage fluidized bed bioreactor system, filled with PVC as the fluidized bed (first stage) and gravel for the filtration purpose (second stage), for the post-treatment of the secondary wastewater treatment plant effluent.

## Materials and methods

### Wastewater sample

The raw wastewater samples were obtained from Shahrak Gharb (Tehran, Iran) wastewater treatment plant effluents. The type of treatment in Shahrak Gharb wastewater treatment plant was activated sludge with surface aerator. Some wastewater characteristics of the samples are given in Table 1. Components of the obtained samples were determined by the procedures described in the Standard Methods [9].

**Table 1:** Characteristics of secondary wastewater treatment plant effluents

**Parameters**	**Minimum**	**Maximum**	**Average**	**SD**^**a**^
5-day Biochemical Oxygen Demand (BOD_5_, mg/L)	16.9	21.5	18.76	1.78
Chemical Oxygen Demand (COD, mg/L)	32	40	35.38	3.61
Total Kjeldahl Nitrogen (TKN, mg/L)	0.69	2.76	1.69	0.797
Total Phosphorus (TP, mg/L)	4.5	4.9	4.75	0.16
Total Solids (TS, mg/L)	556	704.6	640.72	64.18
Turbidity (NTU)	6.2	7.5	6.86	0.559
Electrical Conductivity (EC, μmoss/cm)	730	935	833	81.06
Total Coliform (MPN/100 mL)	22 × 10^3^	175 × 10^3^	115800	61650
Fecal Coliform (MPN/100 mL)	14 × 10^3^	105 × 10^3^	58280	37420
pH	7.2	7.5	7.32	0.13

^a^ Standard deviation.

### Preparation of bacterial culture

Multiple tube fermentation method was used for determination of total coliform and also fecal coliform. The Lactose broth, Brilliant Green and EC broth (Merck, Germany) were used for the preparation of the microbial culture. Microbial culture was prepared according to the procedures described in the Standard Methods [9].

### Bioreactor set-up

**Figure 1:**
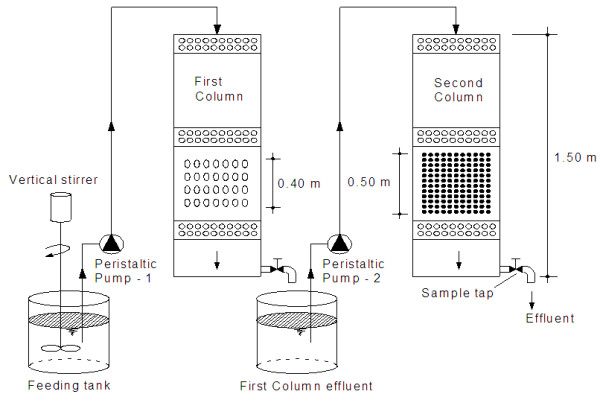
Schematic of the experimental set-up.

A simple schematic of the present bioreactor is depicted in Figure 1. As seen in Figure 1, the FBR system has two cylindrical columns. The first column was filled with PVC material (0.40 m in height) with an effective size of 4 mm and 0.84 g/cm^3^ density. About 40% of the first column was filled with PVC matter for better fluidization of the bed. The second column was filled with gravel (0.50 m in height) with an effective size of 2 mm and 2.5 g/cm^3^ density for the final clarification. The gravels were obtained from the local area. Prior to filling the second bed, the gravels were kept in 10% HCl solution to remove any clay and other residual contaminants. The external diameter, total height and total capacity of both columns were 30 cm, 150 cm and 106 L, respectively. All parts of the reactors were made of transparent Plexiglas material with a wall thickness of 2 mm. The effluents were pumped by peristaltic pumps (Masterflex Cole-Parmer Vernon Hills, IL, USA) at surface loading rates of 5.0, 7.5 and 10.0 m/h. The range of surface loading rates was chosen based on filter media size and target pollutant [10-12]. The FBR system was designed to maintain 2 cm of wastewater column above both beds to ensure a continuous flow rate. Perforated diffuser plates (with 1 mm-hole diameter) were used at the above and bottom of the beds.

On the basis of the preliminary jar test results, aluminum sulfate (30 mg/L) was added into secondary effluents for the coagulation purpose. Chlorine (1 mg/L) was added for the disinfection of the effluents. The wastewater samples were mixed with a vertical stirrer (Lovibond, USA) to obtain a uniform environment in feeding material. The FBR system was operated in a continuous mode feeding by pumping of the fresh feed into the reactors. The bioreactors were maintained at respective temperatures for about 10 days to allow temperature equilibration and the growth of microorganisms. After this period, the performance of the present FBR system was investigated in a temperature-controlled environment (35°C) by collecting wastewater samples from the effluent of the second bed at predetermined intervals of 2 h for the further microbial and physicochemical analyses.

### Pressure drop and washing of beds

The pressure drop was determined as a measure of decrease in flow rate and removal efficiency. The height of the water column above of both columns was maintained as 2 cm to ensure continuous flow rate and to avoid channeling of the wastewater. When the height of the water column reached to about 2.2 cm, the columns were removed and backwashed with the distilled water. Moreover, when the pollutant concentration in bioreactor effluents exceeded the influent concentration, the bioreactor was cleaned by backwashing. In the present work, both methods were applied to investigate the pressure drop problem. The second column was backwashed once per two days of operation to prevent pressure drop. During backwashing of the second bed, the effluent from the first column was re-circulated towards the inlet of the first bed to maintain the microbial activity. For the present case, it was observed that most of the sludge was captured at the internal parts of the second bed.

## Results and discussion

### Removal of BOD_5_ and COD

The concentration of biological oxygen demand (BOD_5_) in wastewater treatment plant effluents can significantly influence the dissolved oxygen rate in receiving water bodies. The daily standard for BOD_5_ in secondary wastewater effluents is limited to be maintained at or below 30 mg/L by authorized organization [7,13]. In addition, the concentration of BOD_5_ in clean water or clean rivers is restricted to as low as 2 mg/L [14]. Therefore, any treatment methods must reduce the BOD_5_ concentration to below 2 mg/L.

**Figure 2:**
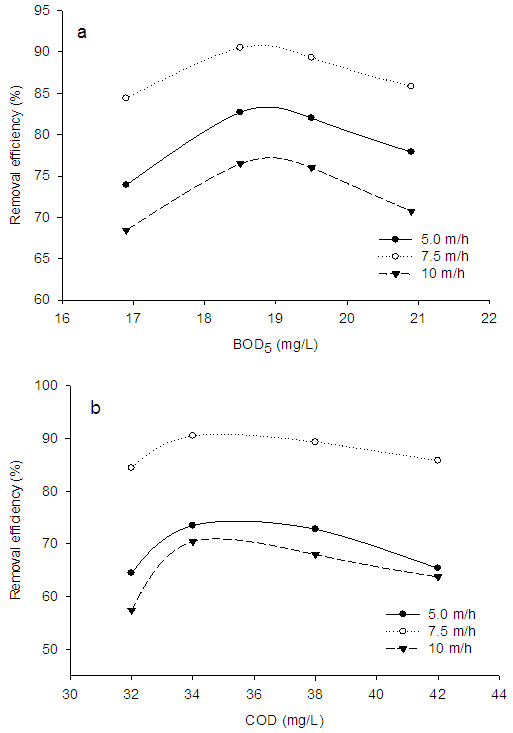
**Removal efficiency of BOD**_**5 **_**(a) and COD (b) at different initial concentrations and surface loading rates.**

Figure 2a shows the removal of BOD_5_ at different surface loading rates. It is clear from this figure that BOD_5_ and surface loading rate significantly influence the removal efficiency. Higher removal efficiency was observed in 7.5 m/h surface rate and 18.5 mg/L of BOD_5_ concentration. In this condition, about 91% of BOD_5_ was removed and the BOD_5_ concentration in the FBR effluent was observed to be 1.67 mg/L. In addition, at that surface rate and the initial BOD_5_ concentration of 19.5 mg/L, the effluent concentration was measured to be about 1.95 mg/L. Therefore, it can be concluded that the present system can meet the available standards at 7.5 m/h of hydraulic rate and the initial BOD_5_ concentration of 18.5 to 19.5 mg/L. The lowest removal efficiency was observed at 20.9 mg/L of BOD_5_ and 10 m/h of hydraulic rate. In this condition, the final concentration of BOD_5_ was determined as 6.11 mg/L.

Figure 2b illustrates the removal of COD at different surface loading rates. Likewise, higher removal efficiency for COD was observed at 7.5 m/h of surface loading rate and initial COD concentration of 34 mg/L. At this condition, about 90.05% of COD was removed and the effluent concentration was reduced to about 3.23 mg/L. For the initial COD concentration of 38 mg/L and at 7.5 m/h of surface loading rate, the effluent concentration of COD was reached to be 4.06 mg/L. Furthermore, the lower removal efficiency was observed at 10 m/h hydraulic rate. Based on the relationship between BOD_5_ and COD [7,9], the present system can reduce the influent COD concentration to below available standards at initial COD concentration of 34 to 38 mg/L and at 7.5 m/h of hydraulic rate.

In the tertiary treatment (coagulation–flocculation–disinfection for irrigation reuse) of a secondary wastewater treatment plant effluent, removal percentages for BOD_5_ and COD (initial BOD_5_ and COD concentrations were 11.6 (± 3.1) mg/L and 38.8 (± 6.3) mg/L, respectively) was obtained to be 46% and 39%, respectively. In the study, the authors reported that the final BOD_5_ and COD concentrations were reached to 6.3 (± 2.4) mg/L and 25.4 (± 4.8) mg/L, respectively [15]. In another work [16], slow sand filtration system was conducted for the post-treatment of up-flow anaerobic sludge blanket (UASB) reactor effluent (average BOD_5_ and COD was about 50 and 120 mg/L, respectively). The study concluded that removal percentages for BOD_5_ and COD were achieved to be 43% and 34% in during the first few hours (36 h) and reached to be about 85% and 79% after 7 days operation, respectively. Therefore, after 7 days operation, the BOD_5_ and COD concentrations in slow sand filter effluent were observed to be 7.5 and 25.2 mg/L, respectively [16]. With respect to slow sand filtration and coagulation–flocculation–disinfection process, results obtained from the present work was remarkable since the final concentrations of BOD_5_ and COD were 1.67 and 3.23 mg/L, respectively, which were much lower than those works.

### Removal of turbidity

**Figure 3:**
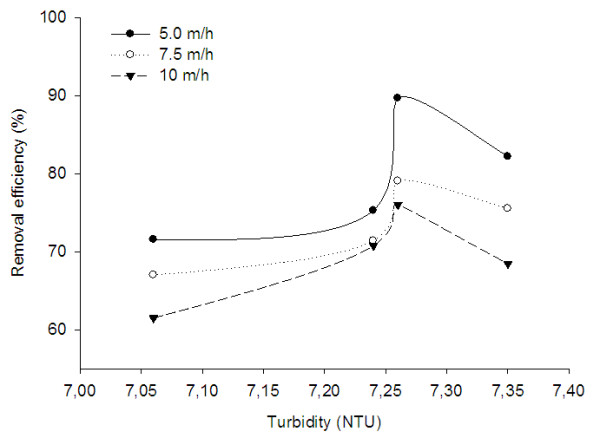
Removal of turbidity at various turbidity unit and surface loading rate.

Removal of turbidity is necessary for an effective disinfection process. In the water environment, the turbidity agent can protect the viral and bacterial organism against the disinfectant matter. For an effective disinfection, the authorized organization have been set up the turbidity standard as low as 1 NTU. In addition, the turbidity can be used as a measure of filter performance and pressure drop [17,18]. For that reason, in this work, removal of turbidity was investigated as an important wastewater characteristic. Figure 3 shows the removal of turbidity at various turbidity unit and surface loading rate. The highest removal efficiency was observed at 5 m/h surface loading rate and 7.26 unit of turbidity. At this condition, about 89.67% of turbidity was removed and therefore final value reached to below 0.75 NTU. As mentioned earlier, for an effective disinfection, turbidity must be lower than 1 NTU. Therefore, the present FBR system is capable to reduce turbidity to below 1 NTU.

Considering the higher percentage removal of turbidity (89.67%) and lower turbidity in the effluent, it can be concluded that the results obtained in the present work was better than the values reported in post treatment of UASB reactor effluent by slow sand filtration [16]. In that work, the average turbidity was 56.5 NTU and the maximum removal percentage was reported to be 91.60%. Based on this removal efficiency, the turbidity in effluent was reached to an average of 2.9 NTU. In that work, the sand depth and effective size was 54 cm and 0.43 mm, respectively. As compared to that work, however higher initial turbidity, the present system containing a fluidized bed with a filter medium with 50 cm gravel depths and 2 mm effective size, seem to be more effective and reliable for the turbidity removal. In another work conducted for the tertiary treatment of municipal sewage via slow sand filtration, higher removal efficiency for turbidity was reported to be about 88% for the sand with an effective size of 0.23 mm and a sand depth of 84 cm [19]. When compared with slow sand filtration [15,19], the present results showed better removal percentage for turbidity. In another work for tertiary treatment of a secondary effluent by the coupling of coagulation–flocculation–disinfection, the initial turbidity was reported to be 6.9 (± 4.3) NTU and after the treatment reached to 1.2 (± 0.4) NTU, which is near to the present results [15]. The overall results clearly demonstrate that only slow sand filtration is not only enough to removal of turbidity and so needs more treatment process such as coagulation or two-stage bed filtration like the present system.

### Removal of total phosphorous

Phosphate exists primarily in the ionized form in the environment and naturally found in some rocks and soils. It is necessary for plant growth as a macronutrient of most of biological being and categorized as one of the main limiting nutrients of organisms living in water resource. The main role of phosphorus in environment is well known as eutrophication in surface water in its excessive limit [20].

**Figure 4:**
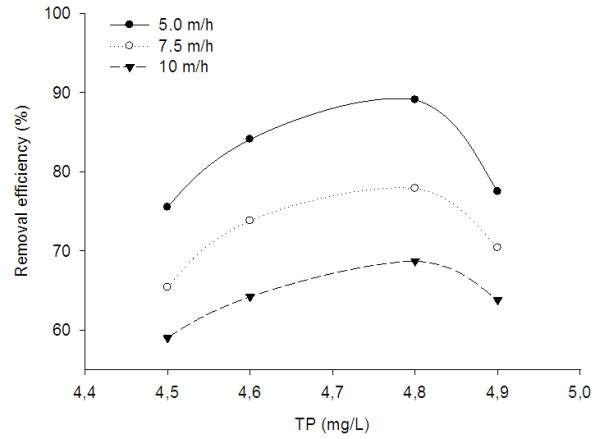
Removal efficiency for total phosphorous at different initial concentration and surface loading rate.

Removal of total phosphorous (TP) by present system is shown in Figure 4. As seen from this figure, removal of phosphorous was increased with the increase in initial phosphorus concentration and reached to its maximum removal efficiency at 4.8 mg/L. In addition, removal efficiency was decreased with the increase in surface loading rate and observed to be maximum at 5 m/h. At the initial phosphorus concentration of 4.80 mg/L and surface loading rate of 5 m/h, removal efficiency was observed to be about 89.1%. The effluent concentration reached to about 0.52 mg/L, which is lower than those reported for the phosphorous concentration (0.5–1.0 mg/L) for protection of the eutrophication [21].

### Coliform removal without chlorination at different organism and hydraulic rate

Water borne diseases are one of the main cases of human illness. Discharge of wastewater treatment plant effluents containing any type of microorganism can cause pollution of water supply resource [22]. On the other hand, improperly designed and operated water treatment plants cannot effectively remove pathogenic organisms from drinking water. This problem can result in nuisance consequences in developing counties where there is no significant water disinfection facilities [18,23,24]. World Health Organization [18] estimated that 1.1 billion people over the world cannot access to safe water. Consumption of contaminated water can cause many severe diseases such as typhoid fever, hepatitis A and E, polio and cholera [5]. Many microorganisms, especially coliform group, can cause various types of waterborne diseases. Therefore, inactivation of pathogenic organisms by appropriate methods is necessary for protection of human health from drinking of contaminated water. Originally, the chlorine and its intermediates are common chemicals that are added into the water to inactivation of pathogenic microorganisms [6,25]. Newly, many researchers indicated that addition of chlorine for disinfection of water can produce many by-products that are carcinogen for human health. Removal of these compounds by an efficient method itself is a serious subject in the field of water disinfection [7]. For that reason, any effective methods such as advanced oxidation processes [26,27] that can be capable to remove pathogenic microorganisms without production of by-products would be valuable. It can be seen from the literature that, the commonly used methods for the microorganism removal are chlorination, ozonation, UV irradiation and other oxidation methods [28]. However, to the best of our knowledge, there is little work for removal of fecal and total coliform with fluidized bed accompanying with a filtration unit. For that reason, in the present work, the efficiency of FBR system with and without chlorination was also investigated for removal of total and fecal coliform organism.

**Figure 5:**
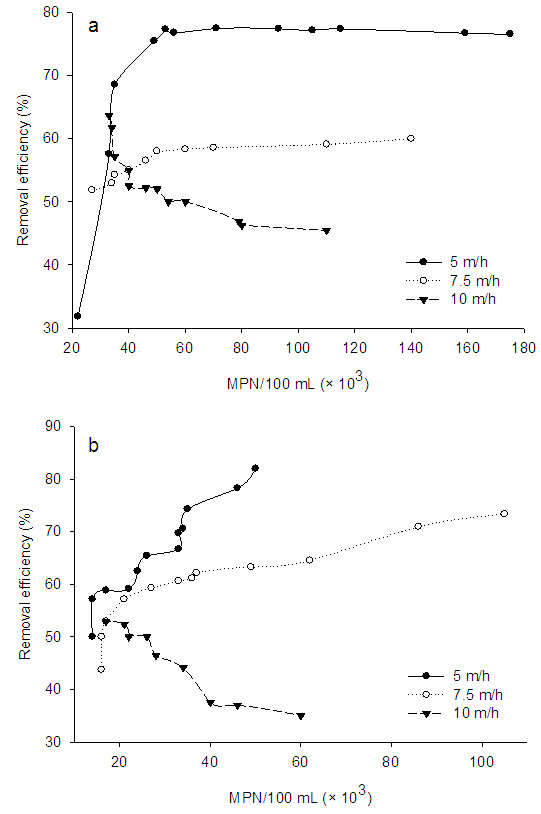
Removal of total coliform (a) and fecal coliform (b) at different microorganism concentration and surface loading rate without chlorination (MPN: Most Probable Number).

Figure 5 shows the removal of total and fecal coliform without any chlorination of effluents at different microorganism concentration and surface loading rate. At the surface loading rate of 5 m/h, removal of total coliform increased as the total coliform concentration increased from 22×10^3^ to 53×10^3^ (MPN, Most Probable Number), and then reached to a stationary stage for increases in total coliform concentration from 53×10^3^ to 175×10^3^ (MPN). For the total coliform concentrations of 22×10^3^, 46×10^3^ and 175×10^3^ (MPN), removal efficiencies were 32, 77 and 77%, respectively. The effluent total coliform concentrations at those removal efficiency values were about 15, 12 and 41×10^3^ (MPN), respectively, which was not acceptable for environmental discharge of effluents without chlorination. Removal of fecal coliform at 5 m/h of surface loading rate increased about linearly with increases in fecal coliform concentration from 14×10^3^ to 50×10^3^ (MPN). Removal efficiencies were 50 and 82% for fecal coliform concentrations of 14×10^3^ and 50×10^3^ (MPN), respectively. According to these removal percentages, the effluent fecal coliform concentrations were determined as 7×10^3^ and 9×10^3^ (MPN), respectively. Results indicated that the final fecal coliform concentrations did not meet the standard levels for the fecal coliform.

At surface loading rate of 7.5 m/h, removal efficiency was increased about linearly for fecal total coliform from 52 to 60% for increases in total coliform concentration from 27×10^3^ to 140×10^3^ (MPN), respectively. For these values, the effluent total coliform concentrations were 13×10^3^ to 56×10^3^ (MPN), respectively. For the fecal coliform, removal efficiency increased from 44 to 73% with increases in coliform concentration from 16×10^3^ to 105×10^3^ (MPN), respectively. The effluent fecal coliform concentration was 9×10^3^ (MPN) at 44% removal efficiency and 28×10^3^ (MPN) at 73% removal efficiency. Result indicated that either total coliform or fecal coliform at those values of removal efficiency did not meet the standard levels for environmental discharges. Consequently, increasing of surface loading rate from 5 to 7.5 m/h led to decreases in coliform removal efficiencies.

At surface loading rate of 10 m/h, total and fecal coliform removal efficiencies decreased with the increase in coliform concentration. With the increase in total coliform concentration from 33×10^3^ to 110×10^3^ (MPN), removal efficiency decreased from 64 to 45% respectively. On the other hand, fecal coliform removal efficiency was decreased from 53 to 35% with the increase in fecal coliform concentration from 17×10^3^ to 60×10^3^ (MPN), respectively. The concentration of total coliform in filter effluents was 12×10^3^ (MPN) at 64% removal efficiency and 60×10^3^ (MPN) at 45% removal efficiency. Similarly, fecal coliform concentration was 8×10^3^ (MPN) at 53% removal efficiency and 39×10^3^ (MPN) at 35% removal efficiency.

Results demonstrated that total and fecal coliform could be removed effectively in surface loading rate of 5 and 7.5 m/h, with increasing in organism concentration. Decreases in coliform removal efficiency at 10 m/h surface rate may be attributed to the removal of biofilm layer from the fluidized bed and also due to disorienting of the filter bed at high hydraulic loading conditions. Therefore, it can be concluded that both surface rate and microorganism concentration will affect the removal efficiency. In overall, the microorganism concentration as coliform from wastewater plant effluent and influent to the FBR system was in the range of 15–175×10^3^ (MPN), and the present system removed about 30% to 83% of microorganism depending on the microorganism type and the surface loading rate. However, it is noted that the discharge standard for secondary wastewater as coliform organism has been reported to be below 200–400 MPN/100 mL [28]. The removal percentage of present system was considerable, but the FBR could not meet the compliance with the effluent discharge standards for the coliform organism without chlorination. Therefore, a proper disinfection will be required when treating secondary wastewater with the FBR system.

### Coliform removal with chlorination

**Figure 6:**
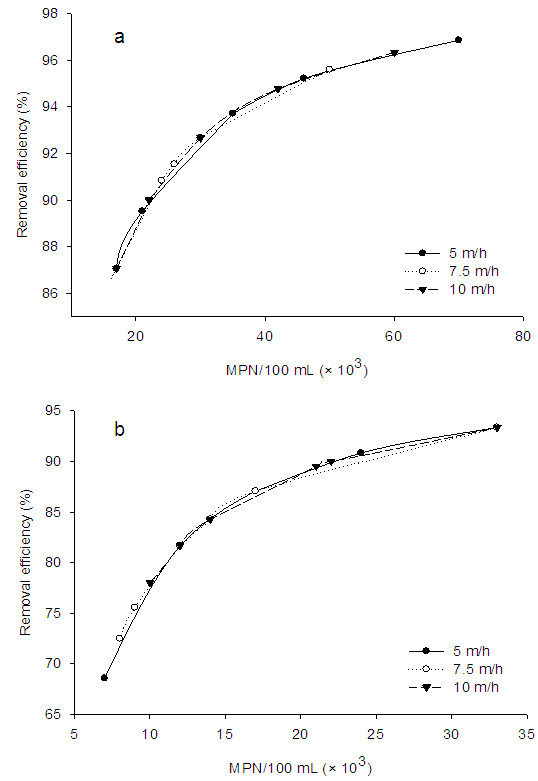
Removal of total coliform (a) and fecal coliform (b) at different microorganism concentration and surface loading rate with chlorination (MPN: Most Probable Number).

Figure 6 shows the effect of chlorine addition on coliform organism removal efficiency in used reactor. As seen from this figure, addition of chlorine leads to increase in microorganism removal without affected by hydraulic loading rate. With chlorination, almost 90 to 98% of both total and fecal coliform were removed depending on coliform concentration and hydraulic surface loading rate. Considering all applied surface loading rates and different microorganism concentrations, the final concentrations for both total and fecal coliform were reached to below 2.2 MPN/100 mL. Therefore, it can be concluded that the FBR system can meet the water standard for drinking water with chlorine addition [13]. It is reported that chlorine concentration for the oxidation of raw or secondary wastewater can be as high as 5–20 mg/L and at such higher level; chlorine addition leads to enhance TS concentration in the treated wastewater [28]. However, in the present work, we observed no increment in TS concentration during FBR–chlorination of secondary wastewater for the removal of coliform organism. More importantly, the used chlorine concentration (1 mg/L) applied to the secondary wastewater was very low as compared to other studies, indicating the advantage of the present system for the microorganism removal.

Table 2 summarizes performance data concerning the comparison of different FBR configurations on treatment of various types of wastewaters such as synthetic starch wastewater [29], pink water [30], real textile wastewater [31], diesel fuel-contaminated wastewater [32], brewery wastewater [33], textile wastewater [34], and high-strength distillery wastewater [35]. The performance data reveals that wide range of operating conditions have been conducted to remove COD, BOD_5_, color, TS, TP and others. Various types of materials such as PVC, gravel, granular activated carbon (GAC), pumice, lava rock particles, small silica particles, polyethylene material and natural zeolite, has been used as growth support media. The performance data figures out that initial pH has been conducted between 6.7 and 7.8, and a wide range of initial COD has been studied in the limits of 32–7000 mg/L. On the basis of maximum removals obtained from different FBR configurations, the present data seems to be comparable with those reported by others (Table 2). However, it is noted that differences are due to the characteristics of studied wastewaters and experimental conditions such as applied loading rates, initial concentration of pollutants, hydraulic retention time, operating temperature, and also different types of support media. These differences may also be attributed to the presence of several recalcitrant inorganic compounds, complex components, and other undesirable impurities in the wastewaters.

**Table 2:** Comparison of different FBR typologies on treatment of various types of wastewaters

**Wastewater type**	**Reactor type and dimensions**	**Support material**	**Initial concentrations and loading rates**	**Operating conditions**	**Efficiency**	**Reference and region**
					**(i.e. COD, BOD**_**5**_**, TS removals, etc.)**	
Secondary wastewater treatment plant effluents	Two-stage FBR, V = 106 L, H = 1.5 m, D = 0.30 m	PVC material and gravel	COD = 32–40 mg/L	pH = 7.2-7.5	90.5%, 91%, 89.7%, 89.1% and 98% of COD, BOD_5_, turbidity, TP and coliform removals, respectively	Present study, Iran
			BOD_5_ = 16.9-21.5 mg/L	T = 35°C		
				v_f_ = 5, 7.5 and 10 m/h		
			TS = 556–704.6 mg/L			
			Turbidity = 6.2-7.5 NTU			
Synthetic starch wastewater	Anaerobic tapered FBR, V = 7.8 L	Granular activated carbon (GAC)	OLR = 1.0-85.44 kg COD/(m^3^.day)	pH = 6.8-7.2	92% of COD removal	Parthiban et al. [3], India
				HRT = 1.97-26.74 h		
			COD = 1100–7000 mg/L			
			BOD_5_ = 690–5960 mg/L			
Pink water	GAC-FBR, H = 4.9 m, D = 0.51 m	GAC	TNT = 3.5-56.2 mg/L	pH = 6.8-7	Effluent TNT = <0.03 – 2.8 mg/L	Maloney et al. [30], USA
				HRT = 125–375 min		
				T = 67–106.5°F		
Real textile wastewater	Anaerobic FBR, V = 4 L, H = 73 cm, D = 5.2 cm	Pumice	OLR = 1–5 kg COD/(m^3^.day) COD = 1030–6000 mg/L	HRT = 24 h	82%, 94% and 59% of COD, BOD_5_ and color removals, respectively	Sen and Demirer [31], Turkey
				T = 35 (±2)°C		
				v_f_ = 19 m/h		
Diesel fuel (DF)-contaminated wastewater	Three-phase FBR, V = 200L, H = 3 m, D = 0.17 m	Lava rock particles	DF = 50–700 mg/L COD = 547–4025 mg/L	pH = 6.7-7.8	>99.9%, 96.2%, 99.9% and 47.8% of DF, COD, TS and turbidity removals, respectively	Lohi et al. [32], Canada
				HRT = 4 h		
				T = 20 (±5)°C		
				v_f_ = 0.3 cm/s		
Brewery wastewater	Anaerobic inverse FBR, V = 1.9 L, H = 1.37 m, D = 4.48 cm	Small silica		pH = 7	>90% of COD removal	Alvarado-Lassman et al. [33], Mexico
		particles and polyethylene material	OLR = 70 kg COD/(m^3^.day)	T = 35°C		
				v_f_ = 6 m/h		
			BOD_5_ = 1375 mg/L			
Textile wastewater	Anaerobic FBR, V = 3.75 L, H = 750 mm, D = 80 cm	Activated carbon	OLR = 1.5-8.4 kg COD/(m^3^.day) COD = 810–4200 mg/L	pH = 7.8	98%, 95% and 65% of COD, BOD_5_ and color removals, respectively	Haroun and Idris [34], Malaysia
				HRT = 4–12 h T = 35°C		
High-strength distillery wastewater	Anaerobic FBR, V = 5.9 L, H = 74 cm, D = 6.5 cm	Natural zeolite	OLR = 3–20 kg COD/(m^3^.day)	pH = 6.7-7.6	>80% of COD removal	Fernandez et al. [35], Chile
				T = 30 (±2)°C		

As seen from the recent literature, there are no systematic papers specifically devoted to a study regarding the application of a two-stage FBR system with a filtration column for the post-treatment treatment of secondary wastewater treatment plant effluents. For this reason, the present study aimed at fulfilling the gap in this field by focusing upon the treatment performance of FBR technology on real secondary effluents. Moreover, most of studies were conducted at laboratory-scale; however, the applicability of the FBR system was specifically investigated at pilot-scale in the present work. Besides conventional waster parameters, the efficiency of a two-stage FBR system was also investigated as a specific objective for inactivation of pathogenic organisms (i.e. total and fecal coliform organism) with and without chlorination. Based on the above-mentioned facts, the novelty of the present study is highlighted with comparison of experimental results from the previous publications.

## Conclusion

The study revealed that a two-stage pilot-scale FBR system for secondary wastewater treatment was technically feasible in terms of BOD_5_, COD, turbidity, TP, total coliform and fecal coliform. Depending on both microorganism concentration and applied surface loading rates, the FBR system could meet the available standards with removal percentages above 90%. The FBR–chlorination system could be used as a promising post-treatment process to improve the quality of the final discharge without any increase in TS concentration during the chlorination.

## Competing interests

The authors declare that they have no competing interests.

## Authors’ contributions

GHS was involved in experimental parts and take the initial samples from wastewater treatment plant. KY was involved in drafting the manuscript or revising it critically for important intellectual content, and made substantial contributions to conception and design. AHM reviewed the final manuscript and also involved in experimental parts. MZ participated in the design of the study, made substantial contributions to acquisition of data, or analysis and interpretation of data, and gave final approval of the version to be published. All authors read and approved the final manuscript.
